# Analysis of Japanese Patients Treated with or without Long-Term Epirubicin Plus Ara-C Intravesical Instillation Therapy for Low-Grade Superficial Bladder Cancer

**DOI:** 10.1155/2015/325305

**Published:** 2015-05-21

**Authors:** Tomonori Kato, Kazushi Nomura, Fukuo Kondo, Masami Wakisaka, Akira Komiya

**Affiliations:** ^1^Department of Urology, Social Insurance Funabashi Central Hospital, 6-13-10 Kaijin, Funabashi, Chiba 273-8556, Japan; ^2^Department of Urology, Graduate School of Medicine and Pharmaceutical Sciences for Research, University of Toyama, 2630 Sugitani, Toyama-shi, Toyama 930-0194, Japan; ^3^Department of Pathology, Social Insurance Funabashi Central Hospital, 6-13-10 Kaijin, Funabashi, Chiba 273-8556, Japan; ^4^Department of Pathology, Teikyo University, 2-11-1 Kaga, Itabashi-ku, Tokyo 173-8605, Japan

## Abstract

The high incidence of tumor recurrence following transurethral resection (TUR) represents a major problem encountered in the management of bladder cancer. This study examined the efficacy of intravesical chemotherapy in superficial bladder cancer. We retrospectively analyzed 90 Japanese cases with low-grade superficial transitional cell carcinoma (stage T1, grades 1 and 2) who were rendered tumor-free by TURBT (TUR of bladder tumor) and who thereafter were treated with or without intravesical chemotherapy. Among them, instillation was terminated in 2 patients due to adverse effects (severe but reversible chemical cystitis). Remaining 88 patients were divided into 2 groups according to therapy: the TURBT-only group (*n* = 46), defined as patients treated with TURBT alone, and the Instillation group (*n* = 42), defined as patients treated with weekly intravesical instillation therapies using epirubicin plus Ara-C. Recurrence-free rate was significantly higher in the Instillation group than in the TURBT-only group (*p* = 0.02, HR = 0.457). The 5-year recurrence-free rate was 58.5% for the Instillation group and 38.6% for the TURBT-only group. Our instillation schedule represents the most intensive regimen among previously reported therapies and resulted in a 54.3% decrease in incidence of tumor recurrence. We believe that the results of this study could provide useful information on management of bladder cancer.

## 1. Introduction

The high incidence of tumor recurrence following transurethral resection (TUR) continues to be a major problem encountered in the treatment of bladder cancer. Intravesical chemotherapy after TUR has been developed to eliminate existing cancer cells and prevent recurrence and progression. Numerous reports have examined the timing and duration of instillation therapy, but no widely accepted optimum treatment regimens have been determined [1–3]. Classically, instillation therapies are categorized as short- or long-term. Recently, the use of immediate postoperative instillations of intravesical chemotherapy after TURBT is also reported [4–6].

In our institution, long-term chemotherapies for stage T1 bladder cancer had been performed between April 1988 and May 2004. Our instillation schedule is the most intensive weekly regimen among previously reported long-term therapies. We retrospectively analyzed data of consecutive 90 Japanese patients treated with or without long-term epirubicin plus Ara-C intravesical instillation therapy for low-grade superficial bladder cancer. This case-control study examined the efficacy of long-term intravesical adjuvant chemotherapy in superficial bladder cancer.

## 2. Materials and Methods

Subjects comprised consecutive 90 Japanese patients with low-grade superficial transitional cell carcinoma (stage T1, grades 1 and 2) who were rendered tumor-free by TUR of bladder tumor (TURBT) and thereafter treated with or without intravesical chemotherapy between April 1988 and May 2004. These 90 patients have no previous history of grade-3 tumor or carcinoma in situ, no history of BCG (Bacillus Calmette-Guerin) instillation therapy, and no coexistence or past history of upper urinary tract transitional cell carcinoma. Patients with other active neoplasms or serious systemic complications were also excluded from analysis. All patients underwent complete TUR of all visible lesions. No patients were submitted to a second TUR before to start with this therapy because, in our institution, second TUR had not been performed in this period. All histological examinations were performed at the Institute of the Pathology at our hospital. Tumors were classified into 3 grades (G1, G2, or G3) according to WHO classifications and stage was determined according to the UICC TNM classification system.

Among them, there were 44 patients treated with intravesical chemotherapy. Decision to undergo treatment with IV was made by attending physician and was made after TURBT. Among them, instillation was terminated due to adverse effects (severe but reversible chemical cystitis) in 2 of 44 intravesically treated patients and excluded from further analysis. Subsequently, the remaining 88 of 90 patients were retrospectively divided into 2 groups according to therapy: the TURBT-only group (*n* = 46), patients treated using TURBT alone; and the Instillation group (*n* = 42), patients treated with post-TUR long-term intravesical instillation therapies using epirubicin plus Ara-C (cytosine arabinoside).

**Figure 1:**
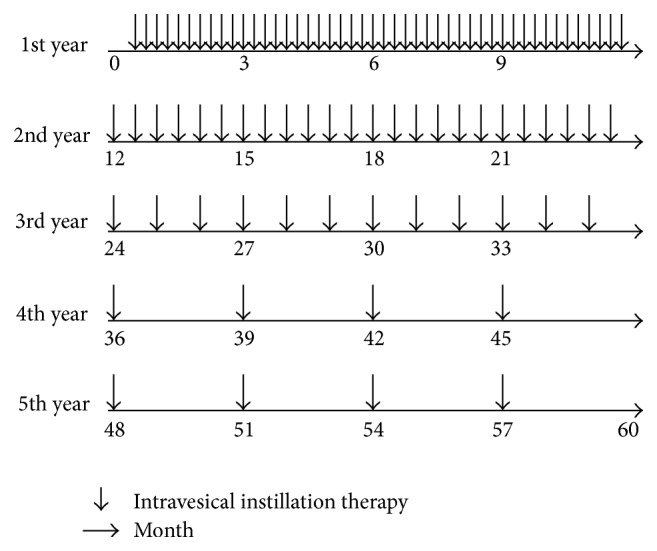
Instillation group was treated using long-term intravesical instillation of 30 mg of epirubicin plus 200 mg of Ara-C dissolved in 20 mL of physiological saline.

The Instillation group was treated using long-term intravesical instillation of 30 mg of epirubicin plus 200 mg of Ara-C dissolved in 20 mL of physiological saline weekly for the first year, then every 2 weeks for the second year, once a month for the third year, and once every 3 months during the fourth and fifth years (Figure 1). Weekly intravesical administration for superficial bladder cancer is the maximum available dosage approved by the Japanese health insurance system. Patients were instructed to retain the solution for 2 hours before voiding.

As follow-up to detect recurrent bladder cancer, cystoscopy was performed every 3 months up to the third year, then every 6 months up to the fifth year, and annually thereafter. Urinary cytological results were also examined once a month during the first year, then every 3 months up to the fifth year, and every 6 months thereafter. Follow-up schedule was the same for both groups. This follow-up schedule is available under the Japanese health insurance system. When recurrences occurred clinically, TURBT was performed and the recurrences were histologically confirmed.

Statistical analysis of data was performed using StatView software (SAS Institute, Cary, NC). The significance of any differences in patient characteristics among groups was tested using Fisher's PLSD, Kruskal-Wallis test, or Mann-Whitney *U* test. Recurrence-free survival of patients was determined using the Kaplan-Meier method, and differences were evaluated using the log-rank test. Values of *p* < 0.05 were considered statistically significant. A Cox regression model was used to isolate independent prognostic factors.

The study conformed to the principles outlined in the Declaration of Helsinki. The Ethics Committee of Social Insurance Funabashi Central Hospital approved this study and waived the approval and informed consent for participation in the study, because of the retrospective nature of this analysis of clinical data. The reference number is H26-6.

## 3. Results

### 3.1. Patient Characteristics

Mean follow-up period was 24.4 months in the TURBT-only group and 33.7 months in the Instillation group. No significant differences were noted between groups in terms of age, sex, previous bladder cancer history, tumor number, growth pattern, or histological grade of tumor. With regard to tumor size, the Instillation group displayed significantly larger tumors than the TURBT-only group (*p* < 0.01) (Table 1).

### 3.2. Recurrence-Free Survival

**Figure 2:**
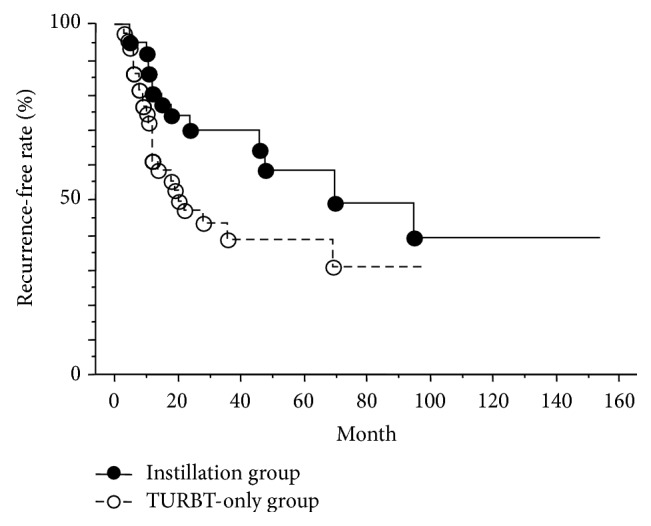
Comparison of recurrence-free survival between Instillation group (●) and TURBT-only group (○). Recurrence-free rate was significantly higher in the Instillation group than in the TURBT-only group (*p* = 0.04). The 1-, 3-, and 5-year recurrence-free rates were 80.6%, 69.7%, and 58.5% for the Instillation group and 61.2%, 43.4%, and 38.6% for the TURBT-only group.

Overall recurrence-free curves for both treatment groups are shown in Figure 2. The 1-, 3-, and 5-year recurrence-free rates were 80.6%, 69.7%, and 58.5% for the Instillation group and 61.2%, 43.4%, and 38.6% for the TURBT-only group. Recurrence-free rate was significantly higher in the Instillation group than in the TURBT-only group (*p* = 0.04).

A Cox regression model was used to isolate independent prognostic factors among sex, age, previous bladder cancer history (yes versus no), tumor grade, tumor multiplicity, tumor size, growth pattern (papillary versus nonpapillary), and treatment protocol (Instillation versus TURBT-only). Previous bladder cancer history, tumor grade, and tumor multiplicity proved to represent independent prognostic factors for local recurrence (odds ratios, 2.41, 2.20, and 2.26; *p* ≤ 0.01, 0.01, and 0.03, resp.). Instillation therapy also significantly reduced relative risk (odds ratio, 0.457; *p* = 0.02). Other factors made no significant contribution to recurrence rate (Table 2).

### 3.3. Disease Progression of Recurrent Tumors

Disease progression was defined as recurrent tumor with muscular invasion, progression of grade to G3, or distant metastasis. No patients displayed recurrence involving distant metastasis. Recurrent G3 cancer was detected in 7 of all 88 patients, comprising 3 of the 46 TURBT-only patients and 4 of the 42 Instillation patients, with no significant differences between groups. Among the 4 patients in the Instillation group with recurrent G3 cancer, 1 patient who demonstrated muscular invasion refused total cystectomy and died from the disease 19 months after disease progression. Recurrent cancer with muscular invasion was detected only in this case and not in any of the other 87 patients.

### 3.4. Adverse Effects

As described above, instillation was terminated in 2 of 44 intravesically treated patients due to severe but reversible chemical cystitis, which occurred 9 and 12 months after initial treatment. In both cases, symptoms were relieved within 2 weeks with observations. The remaining 42 patients were all pursued with the instillation regimen. No myelosuppression, cardiovascular events, renal toxicity, or other systemic adverse effects were identified.

## 4. Discussion

The purpose of intravesical chemotherapy after TUR is to eliminate existing cancer cells and prevent recurrence and progression. Epirubicin, a derivative of doxorubicin, is an anthracycline antibiotic that undergoes minimal transurothelial absorption [7] and is as or more effective but less toxic than doxorubicin in clinical use [1, 2]. Conversely, Ara-C is an antagonist of pyrimidine metabolism and is generally used for leukemia. As bladder instillation agents, effectiveness of Ara-C alone was less than that of mitomycin C and adriamycin, but Ara-C alone demonstrated a lower incidence of local or general adverse effects and combination therapy proved effective [8].

Numerous reports have examined the timing and duration of instillation therapy, but no widely accepted optimum treatment regimens have been determined. Classically, instillation therapies are categorized as short- or long-term. Intravesical chemotherapy may reduce the possibility of tumor cell implantation and microscopic tumor residues in the early period following TUR [3, 4]. However, short-term therapy is still unlikely to influence the mechanism of recurrence development, which is a target of long-term instillation therapy [9].

Koga et al. [10] performed a randomized trial comparing 1-year long-term and 3-month short-term instillations of 30 mg/mL epirubicin and reported that long-term instillation of epirubicin is significantly more effective than short-term instillation. In a meta-analysis of 3703 patients from 11 randomized trials, Huncharek et al. [11] demonstrated that the chemotherapy treatment schedule may account for variations in tumor recurrence rates among different studies, with long-term therapies producing a greater reduction in recurrence rate than short-term schedules. Statistical findings show that clinical effects are dose-related.

Various long-term therapies have been reported previously and frequency of instillation varies from monthly to biweekly. Our treatment schedule represents the most intensive weekly regimen of the long-term therapies reported to date. Our instillation protocol offers a 54.3% decrease in the frequency of tumor recurrence (odds ratio, 0.457). As regards the cohort characteristics, significant differences in tumor size were noted between groups, with larger tumors in the Instillation group. Despite this finding, recurrence-free rate remained significantly higher in the Instillation group than in the TURBT-only group (*p* = 0.04). Some selection bias may have been present due to our positive recommendation for patients with large tumor. However, tumor size was not identified as a significant prognostic factor for development of local recurrence. Previous bladder cancer history, tumor grade, and tumor multiplicity proved to represent independent prognostic factors for local recurrence, as previously reported [12].

To our surprise, impressive decreases of up to 50% in tumor recurrence rate after just 1 immediately postoperative instillation have been reported recently [4–6]. Our results did not have sufficiently superior outcomes to those of single immediately postoperative instillations at a glance. However, our study regards a particular population, patients with T1 G1/G2 cancer. A significant proportion of the population would be reclassified today to T1 high grade cancer using the latest grading system. It should not be compared to early single instillation whose benefits are yet controversial and limited to neoplasms of lowest potential [13].

The limitations of this study include its nonrandomized retrospective design performed at a single center and the relatively small number of patients. The results could also have been biased by a long period of time to accrue 1988–2004. Due to the above mentioned selection bias based on tumor size toward instillation of chemical agents, this study most likely underestimated the benefit of our therapy. Further prospective studies with large populations are needed to clarify this issue. Nevertheless, even with these limitations, the current results would provide useful information on intravesical instillation chemotherapy.

## 5. Conclusions

This study demonstrates the efficacy of long-term instillation of chemical antineoplastic agents in preventing recurrence of superficial low-grade bladder cancer after TURBT. We believe that the results of this study could provide useful information on management of bladder cancer.

## Figures and Tables

**Table 1:** Subject characteristics.

	TURBT-only group	Instillation group	*p *
*n*	46	42	
Age (years)	62.5 ± 11.4	62.8 ± 12.8	0.90
Sex			0.43
Male	35	36	
Female	11	6	
Tumor grade			0.77
G1	17	14	
G2	29	28	
Previous bladder cancer history			0.22
No	27	31	
Yes	19	11	
Tumor multiplicity			0.74
1	38	33	
2–4	7	9	
≥5	1	0	
Tumor size (cm)			<0.01
<1	27	11	
≥1, <3	18	26	
≥3	1	5	
Growth pattern			0.64
Papillary	39	38	
Nonpapillary	7	4	

TURBT: transurethral resection of bladder tumor.

**Table 2:** Univariate and multivariate analysis of significant predictors of tumor recurrence.

Variables	Univariate analysis		*p* value	Multivariate analysis		*p* value
	HR	95% C.I.		HR	95% C.I.	
Age	0.97	0.94–1.01	0.12	0.98	0.95–1.00	0.14
Sex						
Male/female	0.54	0.23–1.23	0.14	0.71	0.35–1.41	0.33
Previous bladder cancer history						
Yes/no	2.81	1.40–5.64	0.01	2.41	1.26–4.59	<0.01
Tumor grade						
G2/G1	1.84	0.91–3.71	0.08	2.20	1.19–4.06	0.01
Multiplicity						
Multiple/solitary tumor	2.88	1.27–6.52	0.05	2.26	1.05–4.82	0.03
Tumor size						
>1/<1	2.11	1.02–4.38	0.06	0.73	0.34–1.58	0.43
Growth pattern						
Nonpapillary/papillary	1.31	0.40–4.16	0.86	1.11	0.41–3.00	0.83
Treatment protocol						
Instillation/TURBT-only	0.44	0.21–0.92	0.04	0.45	0.24–0.85	0.02

TURBT, transurethral resection of bladder tumor; HR, hazard ratio; C.I., confidence interval.

## Abbreviations

TUR: Transurethral resection
TURBT: TUR of bladder tumor
Ara-C: Cytosine arabinoside
BCG: Bacillus Calmette-Guerin.

## References

[1] Badalament R. A., Farah R. N. (1997). Treatment of superficial bladder cancer with intravesical chemotherapy. *Seminars in Surgical Oncology*.

[2] Ali-El-Dein B., El-Baz M., Aly A. N. M., Shamaa S., Ashamallah A. (1997). Intravesical epirubicin versus doxorubicin for superficial bladder tumors (stages pTa and pT1): a randomized prospective study. *The Journal of Urology*.

[3] Tolley D. A., Parmar M. K. B., Grigor K. M. (1996). The effect of intravesical mitomycin C on recurrence of newly diagnosed superficial bladder cancer: a further report with 7 years of followup. *The Journal of Urology*.

[4] Oosterlinck W., Kurth K. H., Schroder F. (1993). A prospective European Organization for Research and Treatment of Cancer Genitourinary Group randomized trial comparing transurethral resection followed by a single intravesical instillation of epirubicin or water in single stage Ta, T1 papillary carcinoma of the bladder. *Journal of Urology*.

[5] Solsona E., Iborra I., Ricós J. V., Monrós J. L., Casanova J., Dumont R. (1999). Effectiveness of a single immediate mitomycin C instillation in patients with low risk superficial bladder cancer: short and long-term follow-up. *Journal of Urology*.

[6] Rajala P., Kaasinen E., Raitanen M., Liukkonen T., Rintala E. (2002). Perioperative single dose instillation of epirubicin or interferon-*α* after transurethral resection for the prophylaxis of primary superficial bladder cancer recurrence: a prospective randomized multicenter study—Finnbladder III long-term results. *The Journal of Urology*.

[7] Cersosimo R. J., Hong W. K. (1986). Epirubicin: a review of the pharmacology, clinical activity, and adverse effects of an adriamycin analogue. *Journal of Clinical Oncology*.

[8] Araki H., Mishina T., Miyakoda K., Fujiwara T., Kobayashi T. (1982). Cytosine arabinoside bladder instillation therapy for bladder tumors. *Tohoku Journal of Experimental Medicine*.

[9] Akaza H., Kurth K. H., Williams R. (1998). Intravesical chemotherapy and immunotherapy for superficial tumors: basic mechanism of action and future direction. *Urologic Oncology*.

[10] Koga H., Kuroiwa K., Yamaguchi A., Osada Y., Tsuneyoshi M., Naito S. (2004). A randomized controlled trial of short-term versus long-term prophylactic intravesical instillation chemotherapy for recurrence after transurethral resection of Ta/T1 transitional cell carcinoma of the bladder. *Journal of Urology*.

[11] Huncharek M., Geschwind J.-F., Witherspoon B., McGarry R., Adcock D. (2000). Intravesical chemotherapy prophylaxis in primary superficial bladder cancer: a meta-analysis of 3703 patients from 11 randomized trials. *Journal of Clinical Epidemiology*.

[12] Ali-El-Dein B., Sarhan O., Hinev A., Ibrahiem E.-H. I., Nabeeh A., Ghoneim M. A. (2003). Superficial bladder tumours: analysis of prognostic factors and construction of a predictive index. *BJU International*.

[13] Perlis N., Zlotta A. R., Beyene J., Finelli A., Fleshner N. E., Kulkarni G. S. (2013). Immediate post-transurethral resection of bladder tumor intravesical chemotherapy prevents non-muscle-invasive bladder cancer recurrences: an updated meta-analysis on 2548 patients and quality-of-evidence review. *European Urology*.

